# The Omentum as a Lymphoid Tissue: Its Molecular Aspects and Application in a Murine Model

**DOI:** 10.1055/a-2832-6664

**Published:** 2026-05-29

**Authors:** Masahiro Hojo, Dongkyung Seo, Riri Ito, Kosuke Ishikawa, Takahiro Miura, Emi Funayama, Yuhei Yamamoto, Taku Maeda

**Affiliations:** 1 Department of Plastic and Reconstructive Surgery, Faculty of Medicine and Graduate School of Medicine, Hokkaido University, Sapporo, Hokkaido, Japan

**Keywords:** omentum, lymphedema, hindlimb

## Abstract

**Background:**

The omentum is a vascularized, immune-active tissue with regenerative potential, particularly when activated by intraperitoneal stimuli. Its secreted factors may promote lymphangiogenesis, offering a novel approach to lymphedema treatment.

**Methods:**

The omentum was activated in mice using an intraperitoneal polydextran slurry. Gene expression was evaluated over time to assess inflammatory and lymphatic markers. Culture supernatants from the activated omentum were collected, and vascular endothelial growth factor-C ( *VEGF-C* ) levels were measured. Therapeutic potential was tested in a mouse hindlimb lymphedema model through local application of the supernatant. Histological analysis assessed skin thickness, lymphatic vessel density, and macrophage infiltration.

**Results:**

Activation of the omentum induced early upregulation of hypoxia-inducible factor-1 α ( *HIF-1α* ) and angiopoietin-2 ( *Ang2* ), followed by increased expression of Forkhead Box C2 ( *Foxc2* ) and Prospero homeobox-1 ( *Prox1* ) by day 7, indicating lymphatic maturation. *VEGF-C* levels in the supernatant were significantly elevated. In the lymphedema model, treated mice exhibited reduced peak edema, faster resolution, and increased skin thickness and lymphatic vessel density compared with controls. Histological analysis revealed enhanced lymphangiogenesis and reduced macrophage infiltration. Downregulation of lymphatic vessel endothelial hyaluronan receptor 1 ( *Lyve1* ) and Neuropilin-1 ( *NRP1* ) suggested a predominance of tissue remodeling over structural maintenance.

**Conclusions:**

Activated omental tissue secretes potent bioactive factors that promote lymphangiogenesis and tissue regeneration. These findings indicate the potential of activated omentum for functional characterization and possible applications in regenerative medicine. Further investigation in chronic and clinical models is warranted to advance its translational potential.

## Introduction

Lymphedema is a chronic condition characterized by the accumulation of lymphatic fluid in tissues, which leads to swelling, pain, and impaired mobility. Current treatments primarily focus on symptom management and do not address the underlying lymphatic dysfunction. Surgical interventions for lymphedema include lymphovenous anastomosis, lymph node transfer, and omental flap transfer. The greater omentum, in particular, has been used in lymphedema treatment since 1966. 1 Omental flaps have also been widely employed as effective surgical interventions for lymphedema, providing a structural scaffold and rich bioactive molecules that support lymphatic drainage and regeneration, and significantly promote lymphatic reconnection and improve drainage. 2 3 Given their rich vascular network, abundant immune cells, and regenerative potential, omental flaps are considered a potent therapeutic option for severe cases of lymphedema. 4 However, concerns regarding donor site morbidity and the invasiveness of harvesting procedures have prompted the development of less invasive techniques, such as laparoscopic or partial harvesting. 2 5

The omentum is a large fatty structure in the abdominal cavity that possesses remarkable healing properties and is rich in blood vessels, immune cells, and progenitor cells. 6 Activation of the omentum, whether via stimulation with polydextran particles, biological modifiers, or changes in the peritoneal microenvironment, enhances its regenerative capabilities by expanding milky spots, increasing vascularization, and recruiting immune and progenitor cells. 6 7 8 Animal studies have demonstrated the regenerative potential of activated omentum in various tissues, such as the skin, cardiac and skeletal muscles, nerves, and organs, including the liver, pancreas, and kidneys. 9 10 11 12 13 14 Over the past two decades, applications for the omentum have evolved from the direct use of omental tissue to cultured stromal cells and, more recently, to the use of the supernatant derived from cultures of omental tissue. These diverse applications highlight the capacity of the omentum to secrete growth factors and cytokines that drive tissue repair and regeneration across various organ systems. 6 7 8

The purpose of the present study was to elucidate the fundamental potential of culture supernatants derived from activated omentum in promoting lymphangiogenesis. Although it is well-established that the omentum is activated by inflammation and infection in clinical settings, we evaluated the effects of the experimentally induced activation of the omentum in an animal model. Our aim was to provide fundamental insights that may contribute to a broader understanding of the functional role of the omentum and its potential applications in regenerative medicine.

## Methods

### Ethics Statement

All animal experiments and associated procedures conducted in this research were approved by the Institutional Animal Care and Use Committee of our institution (approval number 23-0133).

### Animals

Eight-week-old male C57BL/6N mice were obtained from SLC (Tokyo, Japan) and housed in a controlled environment at 24 °C on a 12-hour light/dark cycle with free access to food and water. All animals underwent a 1-week adaptation period before the experiments.

### Omental Activation and Harvesting

**Fig. 1:**
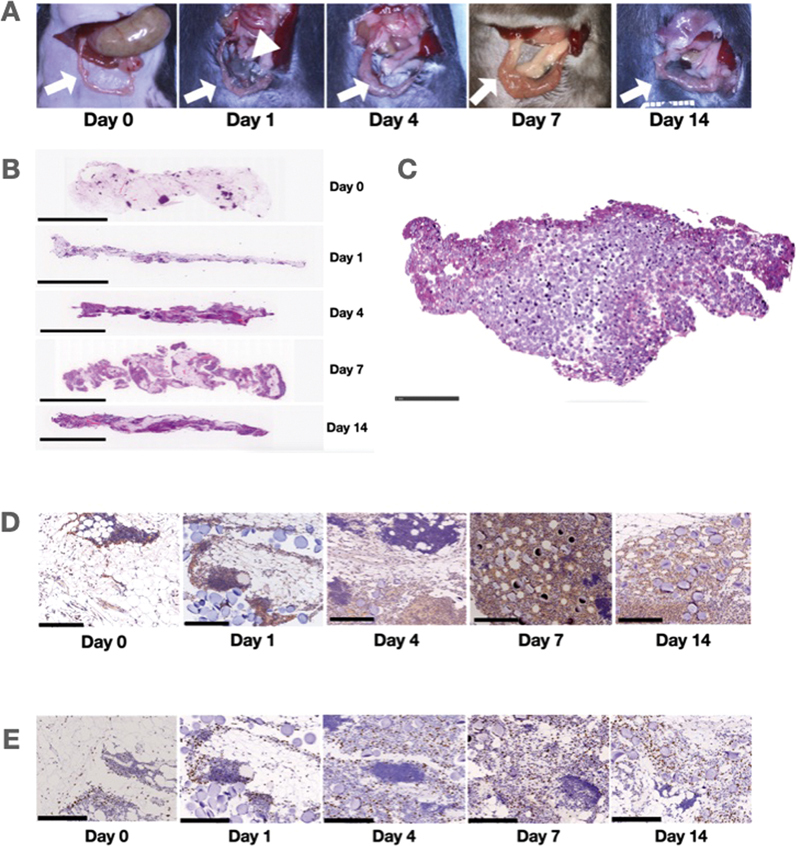
Progression of omental activation. ( **A** ) Sequential photographs of the activation process. Gross findings suggested that the omentum significantly expands in volume and vascularity during the activation process. On day 1, the particle mass is condensed and enclosed by a thin membrane. *(* Arrow indicates omentum; arrowhead indicates particle). ( **B** ) Sequential histological changes (hematoxylin and eosin staining). Histological analysis revealed a gradual decrease in the proportion of adipose tissue and an increase in cellular components. (Scale bar, 5 mm). ( **C** ) Particle surrounded by macrophages (hematoxylin and eosin staining). On day 1, particles began to be entrapped, and the condensed particle mass was surrounded by macrophages. (Scale bar, 1 mm). ( **D** ) Sequential histological changes ( *VEGF-C* staining). On day 0, interstitial spaces were stained, and these spaces expanded during the activation process. (Scale bar, 250 μm). ( **E** ) Sequential histological changes ( *WT-1* staining). *WT-1* -positive cells were observed on day 0, and their numbers increased throughout the process. (Scale bar, 250 μm). *VEGF-C* , vascular endothelial growth factor-C; *WT-1* , Wilms tumor-1.

The mice were randomly divided into three groups. All mice were then injected intraperitoneally with 1 mL of a slurry of polydextran particles (Biogel P-60, 120 μM; 1:1 in normal saline; Bio-Rad Laboratories, Richmond, CA). 15 Seven days later, six mice from each group were euthanized, and the entire omentum was harvested via laparotomy ( Fig. 1 ). The subsequent procedures were conducted in accordance with the steps described in Table 1 .

**Table 1:** Timeline of the procedures performed in each experimental group following intraperitoneal drug administration (day 0)

Day	0 (drug administration)	1	4	7	14
Procedure					
Tissue collection	○	○	○	○	○
Assessment of mRNA quantity	○	○	○	●	○
Tissue collection for culture supernatant preparation				●	

○ = procedure performed in the AO group only.

● = procedure performed in all three groups.

### RNA Extraction from the Omentum and Real-Time Polymerase Chain Reaction Assay

Four mice were euthanized on days 0 (drug administration), 1, 4, 7, and 14 after the injection of polydextran particles. Omental RNA was extracted using an RNeasy Lipid Tissue Mini Kit (Qiagen, Hilden, Germany) and quantified using a DS-11 spectrophotometer (DeNovix Inc., Wilmington, DE). The extracted RNA was then converted to cDNA using a High-Capacity RNA-to-cDNA kit (Applied Biosystems, Foster City, CA). Following conversion, real-time polymerase chain reaction (PCR) assays were performed using a Power SYBR Green PCR Master Mix and a StepOnePlus Real-Time PCR System (Applied Biosystems). The relative levels of PCR products were evaluated using the double delta Ct method. 16 The primers used in this process are listed in Table 2 .

**Table 2:** Primer sequences used in reverse-transcription polymerase chain reaction

Gene	Direction	Sequence (5′–3′)
*Ang2*	Forward	GACTTCTAAGTGGTGGGCACAG
	Reverse	TGAACATGTAAAGCACCCAAGAG
*HGF*	Forward	TCCATGTGGGACAAGAATATGGAG
	Reverse	CATCATCAGGATTCCCAGTA
*HIF-1* α	Forward	TGCGTGCATGTCTAATCTGTTCC
	Reverse	AAGATTCTGACATGCCACATAGCTC
*Foxc2*	Forward	AACGAGTGOGGATTTGTAACCAG
	Reverse	TTGGCAGTAACAGTTGGGCAAG
*Lyve1*	Forward	TCATGCACACCTTAGACACTTAGCC
	Reverse	TGCTTGTCAGCATGCTGTCC
*NRP1*	Forward	AGTCGTTCGAAGGCAACAACA
	Reverse	AGCCCAGTAGCTCCATCCTCA
*NRP2*	Forward	TGGTGATGACAATGGCTGGAC
	Reverse	GCAATGGCTGTGAGCATGG
*Prox1*	Forward	AGCCAGTGTTTAATCTTTGCATCC
	Reverse	AACCATTTGCCTGCTCATTCC
*VEGF-C*	Forward	TGCTGCTGCACATTATAACACAGA
	Reverse	CGGACACACATGGAGGTTTAAAGA
*VEGF-D*	Forward	GCTGCCTGGAAACAACTGCTTA
	Reverse	CCTGAAGCCCTGCACCAAGTA

Abbreviations: *Ang2* , angiopoietin-2; *Foxc2* , Forkhead Box C2; *HIF-1α* , hypoxia-inducible factor-1 α; *Lyve1* , lymphatic vessel endothelial hyaluronan receptor 1; *NRP1/2* , Neuropilin-1/2; *Prox1* , Prospero homeobox-1; *VEGF-C/D* , vascular endothelial growth factor-C/D.

### Collection of the Supernatant

The supernatant was collected using an established method. 9 Briefly, each harvested omentum was rinsed three times with sterile, ice-cold phosphate-buffered saline (PBS), placed in a 12-well plate, and immersed in 2 mL of Dulbecco's Modified Eagle Medium/Nutrient Mixture F-12 supplemented with 10% fetal bovine serum and 1% antibiotic/antimycotic solution. Each plate was then cultured in a 5% CO _2_ atmosphere at 37 °C. After 48 hours of culture, the supernatant was collected and centrifuged twice at 14,800 rpm for 10 minutes at 4 °C. The supernatant was then rapidly frozen using liquid nitrogen and stored at −80 °C to prevent degradation.

### Enzyme-Linked Immunosorbent Assay

The supernatant collected from the omentum was divided among three groups: An activated omentum (AO) group, a native omentum (NO) group, and a PBS-injected (PO) group. Supernatant collected from six mice was used in each group. The concentrations of vascular endothelial growth factor ( *VEGF* )- *C* in the supernatant were determined using a mouse *VEGF-C* enzyme-linked immunosorbent assay (ELISA) kit (Elabscience, Houston, TX) according to the manufacturer's instructions.

### Mouse Model of Lymphedema

A murine model of hindlimb lymphedema was developed using methods detailed elsewhere. 16 Briefly, the procedure involved creating a circumferential skin incision in the left inguinal region, followed by excision of the inguinal lymph nodes and adjacent fat tissue. The pre-nodal and post-nodal lymphatic vessels were ligated before removal of the popliteal lymph nodes and the surrounding fat. A 1-mm-thick silicone sheet was then fashioned into a 3-mm-wide rectangular splint, which was inserted into the inguinal wound and secured to both the skin and underlying muscle tissue.

### Administration of Supernatant

The mice were randomly divided into three groups: The first group received a 0.1-mL subcutaneous injection of AO supernatant ( *n* = 6); the second group received a 0.1-mL subcutaneous injection of NO supernatant ( *n* = 6); and the control group received a 0.1-mL subcutaneous injection of the culture medium used for omental culture ( *n* = 6). These injections were administered on alternate days up to postoperative day (POD) 14, starting on the day of surgery, and all were administered distal to the silicone splint.

### Assessment of Edema

Lymphedema development in the hindlimb was quantitatively evaluated on PODs 0, 2, 4, 6, 8, 10, 12, 14, 17, 21, 24, 28, and 35 ( *n* = 6 per group). The circumference of the hindlimb, 5 mm proximal to the heel, was measured on both sides. The circumference ratio was calculated using the following formula: Circumference ratio = (Treated hindlimb circumference/Untreated hindlimb circumference) × 100%. 17 18

### Histological Assessment

Mice were euthanized on POD 6, and skin samples were harvested. To minimize the influence of inflammation associated with wound healing, samples were collected 6 mm distal to the silicone splint. The skin specimens were fixed in 4% paraformaldehyde, embedded in paraffin, and sectioned for histological analysis.

Skin thickness was evaluated using Elastica–Masson staining. Lymphatic vessel formation and macrophage infiltration were assessed using immunohistochemical staining. For macrophage detection, sections were incubated overnight with anti-F4/80 antibodies (Cedarlane Cold Chain Solutions, Burlington, Ontario, Canada). For lymphatic vessels, anti-podoplanin antibodies (clone D2-40; Abcam, Cambridge, United Kingdom) were used. Histofine (Nichirei, Tokyo, Japan) served as the secondary antibody, and staining was visualized using 3,3-diaminobenzidine (DAB).

Digital images of stained slides were acquired using a whole-slide scanner (NanoZoomer Digital Pathology; Hamamatsu Photonics, Hamamatsu, Japan) and visualized with NDP.view2 software (Hamamatsu Photonics). F4/80-positive and podoplanin-positive areas were quantified using ImageJ software (National Institutes of Health, Bethesda, MD) by analyzing randomly selected fields of view at 100× magnification. Skin thickness was measured from the epidermis to the dermal–fat junction at five randomly selected points per sample, also using ImageJ.

### Statistical Analysis

Data are presented as the mean ± standard error. The significance of differences among the three groups was examined using one-way analysis of variance followed by the Tukey–Kramer multiple comparisons test. All statistical analyses were performed using JMP software (ver. 17.0.0; SAS Institute Inc., Cary, NC). A *p* -value of <0.05 was considered statistically significant.

## Results

### Temporal Changes in Activated Omental Tissue

Following activation, the omentum gradually expanded and exhibited increased capillary density, peaking at day 7. By day 14, the tissue remained mildly dilated compared with baseline ( Fig. 1A ).

### Sequential Histological Changes in Activated Omental Tissue

Histological analysis revealed reduced adipose tissue content and increased cellular components starting from day 1 ( Fig. 1 ). In addition, the administered particles were phagocytosed by macrophages ( Fig. 1C ). Immunostaining for *VEGF-C* and Wilms tumor-1 ( *WT-1* ) was performed to evaluate molecular changes in the activated omentum over time. Regarding *VEGF-C* immunostaining, positive signals were primarily observed in the interstitial spaces on day 0. The signal became more prominent as activation progressed, suggesting expansion of the interstitial compartment and its possible involvement in lymphangiogenic remodeling ( Fig. 1D ). *WT-1* -positive cells were detected on day 0, and their numbers progressively increased throughout the activation period, indicating enhanced mesothelial cell activity or proliferation ( Fig. 1E ).

### Quantity of Omental mRNA

**Fig. 2:**
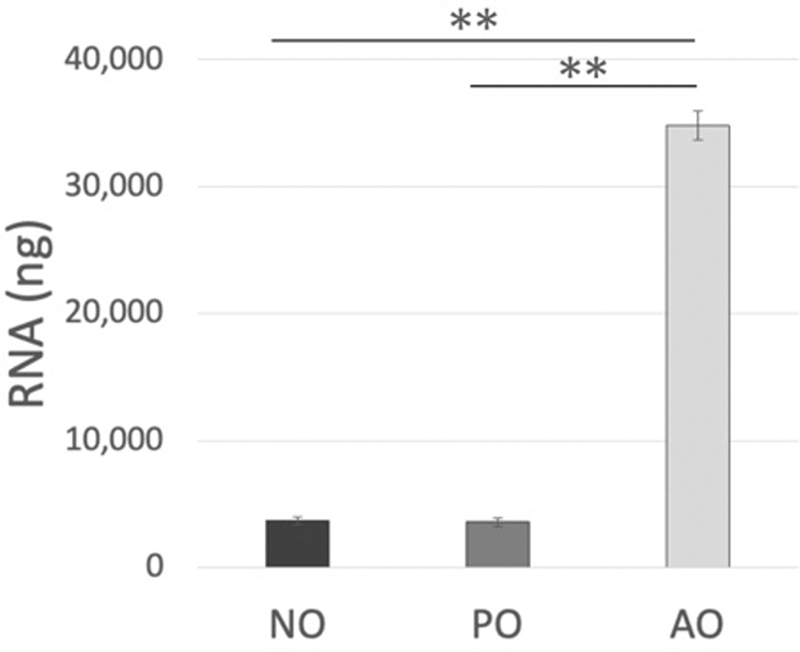
Genetic evaluation of the omentum. mRNA quantity in omental specimens on day 7 ( *n* = 4). mRNA expression was significantly elevated in activated omentum, whereas PBS injection did not induce activation. AO, activated omentum group; NO, native omentum group; PBS, phosphate-buffered saline; PO, PBS-injected group (** *p* <0.01).

The mean RNA expression was nearly 10-fold higher in the AO group (35.49 ± 2.71 μg) than in the NO group (3.82 ± 0.32 μg) and the control group (3.52 ± 0.33 μg; Fig. 2 ).

### Gene Expression in Whole Omentum

**Fig. 3:**
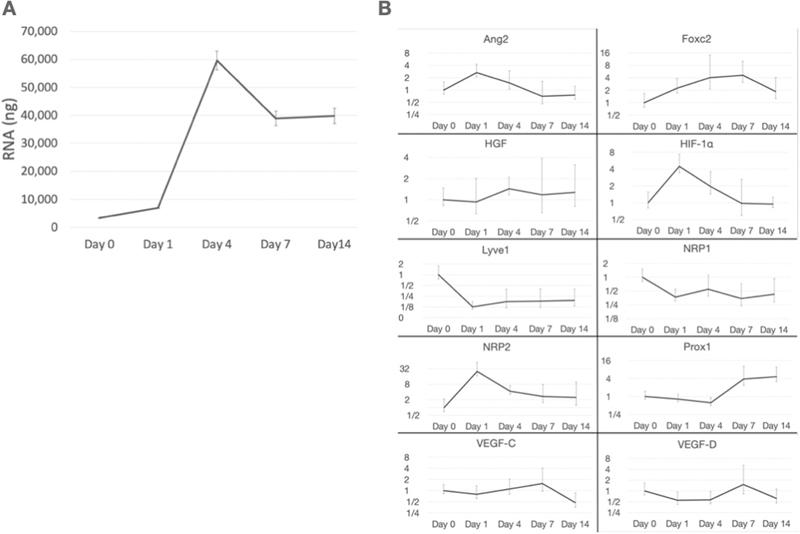
Genetic evaluation of the activated omentum over time. ( **A** ) mRNA quantity over time. mRNA expression showed an exponential increase during the initial days, followed by a gradual decline and plateau after the first week. ( **B** ) Gene expression levels over time for various markers related to lymphangiogenesis. Various markers involved in lymphangiogenesis and tissue regeneration were measured on days 0, 1, 4, 7, and 14. The expression of each gene was normalized, with values indicating relative changes in expression levels across time points. Overall, the data indicated varying expression profiles for each gene, suggesting different roles and activation times in response to tissue stimulation or injury. Early peaks in markers such as *HIF-1α* and *NRP2* may indicate an immediate hypoxic or vascular response, while sustained increases in markers such as *Prox1* suggest ongoing lymphatic or regenerative processes. The figure provides insights into the temporal dynamics of gene expression related to tissue remodeling and lymphangiogenesis. (y-axis, fold change). *Ang2* , angiopoietin-2; *Foxc2* , Forkhead Box C2; *HIF-1α* , hypoxia-inducible factor-1 α; *Lyve1* , lymphatic vessel endothelial hyaluronan receptor 1; *NRP2* , Neuropilin-2; *Prox1* , Prospero homeobox-1; *VEGF* , vascular endothelial growth factor.

mRNA expression exponentially increased between days 1 and 4 after injection and decreased thereafter ( Fig. 3A ). We also evaluated the progression of gene expression associated with lymphangiogenesis ( Fig. 3B ). Neuropilin-2 ( *NRP2* ), angiopoietin-2 ( *Ang2* ), and hypoxia-inducible factor-1 α ( *HIF-1α* ) were increased on day 1, whereas Forkhead Box C2 ( *Foxc2* ) and Prospero homeobox-1 ( *Prox1* ) were increased after day 7. The expression levels of *VEGF-C* and *VEGF-D* , which are considered key genes related to lymphangiogenesis, were not significantly increased. Similarly, hepatocyte growth factor, along with other growth factors such as *VEGF-C* and *VEGF-D* , remained relatively stable, without notable increases. The expression of lymphatic vessel endothelial hyaluronan receptor 1 ( *Lyve1* ) decreased during the omental activation process.

### Vascular Endothelial Growth Factor-C in Supernatant

**Fig. 4:**
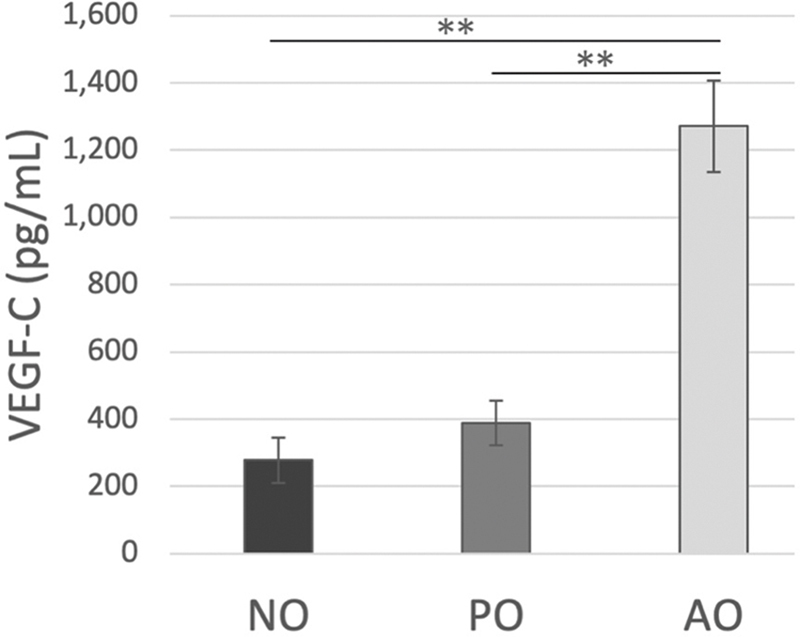
*VEGF-C* levels in supernatant assessed using ELISA. The levels of *VEGF-C* were significantly higher in the AO group compared with the NO and PO groups. During the culture process, *VEGF-C* is believed to be secreted into the supernatant. AO, activated omentum group; ELISA, enzyme-linked immunosorbent assay; NO, native omentum group; PO, PBS-injected group; *VEGF* , vascular endothelial growth factor (** *p* <0.01).

The mean *VEGF-C* level was significantly higher in the AO group (1,270.8 ± 136.0 pg/mL) than in the NO (277.5 ± 68.4 pg/mL) and PO (388.0 ± 65.8 pg/mL) groups, suggesting a substantial increase in *VEGF-C* secretion in the AO group ( Fig. 4 ).

### Gross Assessment of Edema

**Fig. 5:**
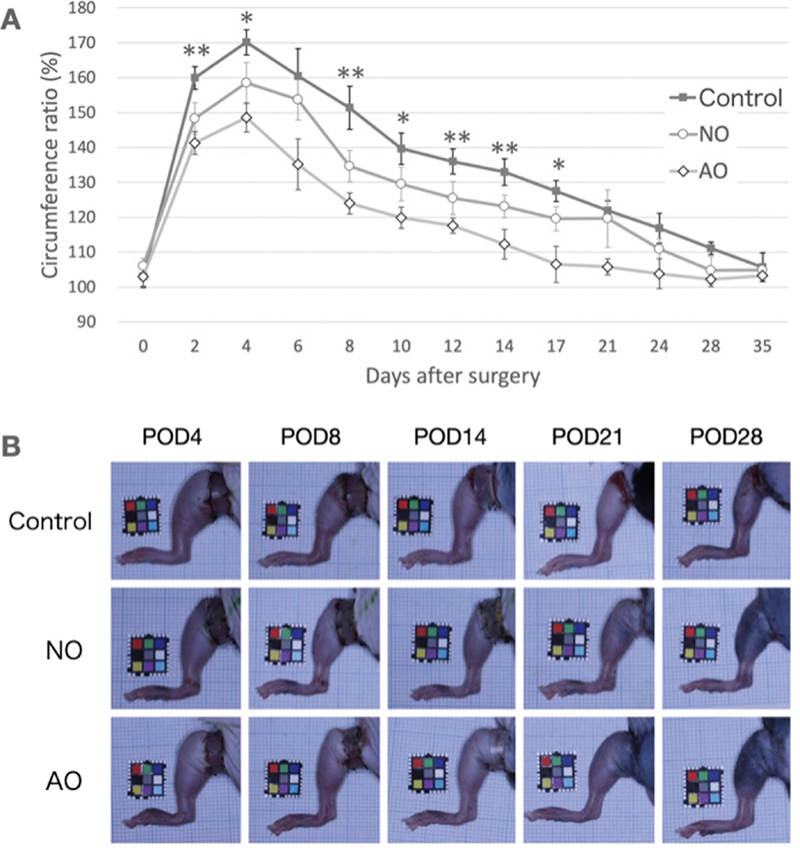
Lymphedema course. ( **A** ) Gross progression of lymphedema. In all groups, the lymphedema peak occurred on day 4 and gradually regressed over the following weeks. Significant differences were observed on PODs 2, 4, 8, 10, 12, 14, and 17 ( *p < 0.05, ** *p* < 0.01). ( **B** ) Representative images of individuals. The groove around the Achilles tendon was more prominent in the AO and NO groups compared with the control group on day 8. AO, activated omentum group; NO, native omentum group; POD, postoperative day.

The circumference ratio peaked on POD 4 in all groups and gradually approached the normal ratio by POD 35. The circumference ratio was significantly lower in the AO supernatant group than in the NO supernatant and control groups on PODs 2, 4, 8, 10, 12, 14, and 17, indicating reduced edema. Figure 5 shows the progressive reduction in edema over 35 days. The decrease in the circumference ratio was more rapid and pronounced in the AO group, suggesting better resolution of edema in this group than in the other groups.

### Histological Findings

**Fig. 6:**
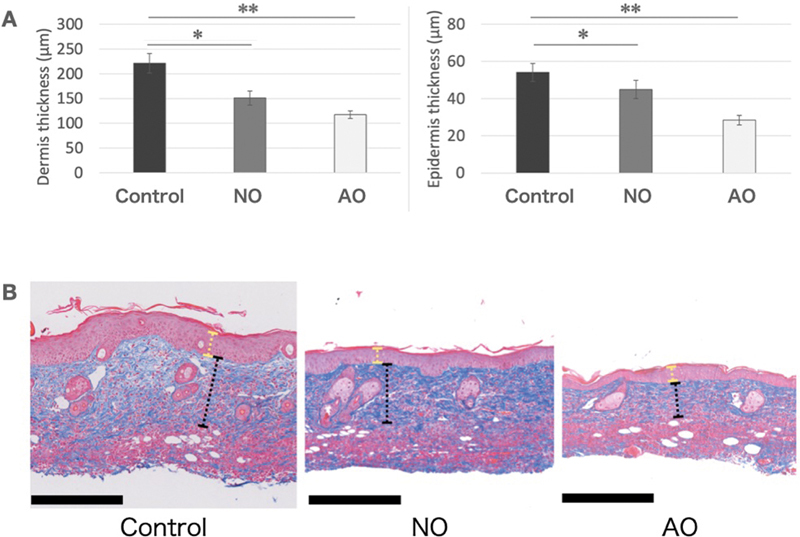
Assessment of skin thickness. ( **A** ) Thickness of the dermis and epidermis. Elastica–Masson staining delineated the skin layers, revealing significant reductions in the thickness of both the dermis and epidermis in the NO and AO groups compared with the control group (* *p* <0.05; ** *p* <0.01). ( **B** ) Histological assessment of skin thickness. Skin thickness was significantly reduced in the AO group. (Yellow dotted line indicates the epidermis; black dotted line indicates the dermis; scale bar, 250 μm). AO, activated omentum group; NO, native omentum group.

The mean dermal thickness was greater in the control group (221.4 ± 19.97 μm) than in the NO (151.03 ± 14.46 μm) and AO (117.65 ± 7.79 μm) groups. A similar trend was observed in the epidermis: The mean epidermal thickness was greatest in the control group (54.06 ± 4.85 µm), followed by the NO group (44.94 ± 4.92 μm) and the AO group (28.37 ± 2.59 μm; Fig. 6 ).

**Fig. 7:**
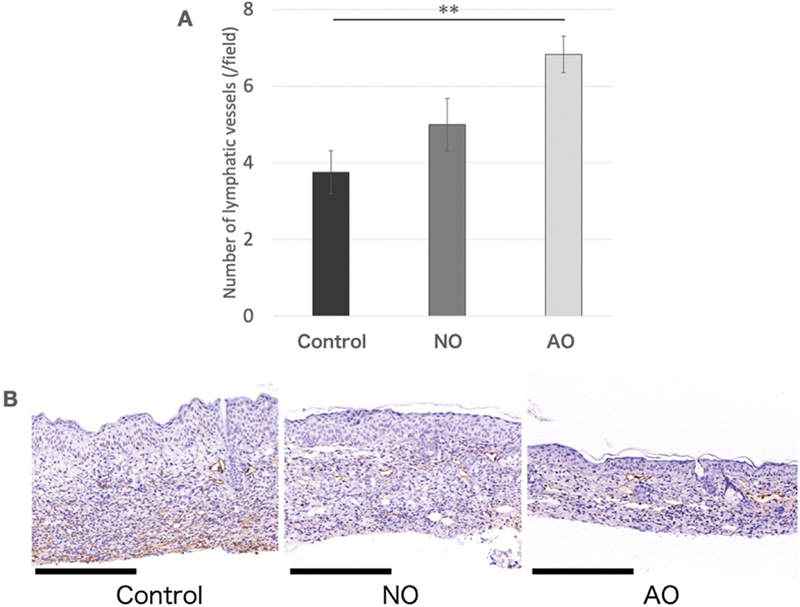
Assessment of lymphatic vessels. ( **A** ) Number of lymphatic vessels. In the AO group, the number of lymphatic vessels was increased, indicating lymphangiogenesis (** *p* <0.01). ( **B** ) Histological assessment of lymphatic vessels. Podoplanin staining highlights the lumens of lymphatic vessels. (Scale bar, 250 μm). AO, activated omentum group.

The mean number of lymphatic vessels was significantly greater in the AO group (6.83 ± 0.48/field) than in the control group (3.75 ± 0.56/field). Although the mean increase in the NO group (5.00 ± 0.68/field) was not significant, the NO was still considered to exert a positive effect on the lymphatic vessel count ( Fig. 7 ).

**Fig. 8:**
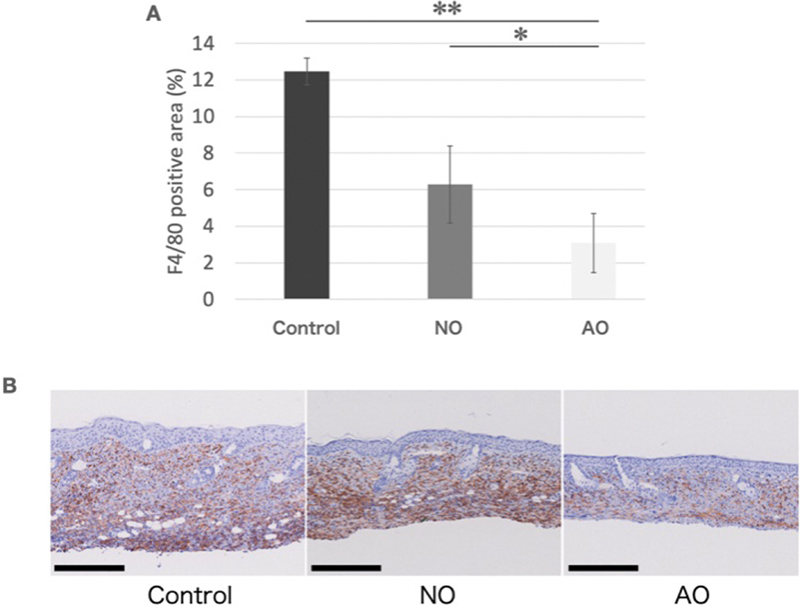
Assessment of macrophage infiltration. ( **A** ) Area of macrophage infiltration. Macrophage infiltration in the layer above the dermoadipose junction was significantly reduced in the AO group. ( **B** ) Histological assessment of macrophage infiltration. F4/80 staining revealed reduced macrophage infiltration in the AO group. (Scale bar, 250 μm). AO, activated omentum group; NO, native omentum group.

The F4/80-positive macrophage area was significantly greater in the control group (12.46% ± 0.72%) than in the NO (6.28% ± 2.10%) and AO (3.08% ± 1.62%) groups, indicating a markedly reduced number of macrophages in the AO group. This reduction potentially reflects differences in the immune response or tissue conditions among the experimental groups. The F4/80-positive area was smaller in the NO group than in the control group, suggesting lower macrophage infiltration in response to treatment with activated or native omentum ( Fig. 8 ).

## Discussion

The regenerative capacity of the omentum has long been a subject of investigation, as demonstrated by the wide range of animal studies presented in Table 3 . These studies highlight the versatility of the omentum across numerous tissues, reflecting its positive role in both wound healing and tissue regeneration. Historically, research has employed various methods for the application and activation of the omentum to enhance its regenerative effects. This approach has evolved from direct tissue application, which closely resembles traditional clinical practice, to less invasive methods such as the use of cultured stromal cells and supernatants derived from omental tissues.

**Table 3:** Animal research into the regenerative therapeutic use of the omentum

Study	Year	Animal	Evaluated organ or tissue	Type of induced injury/engraftment	Material from activated omentum	Induced activation	Application
Marques et al 33	2002	Rat	Spleen	Traumatic	Tissue	No	Mechanical contact
Lee et al 34	2003	Rat	Isolated hepatocyte	Engraftment	Tissue	No	Mechanical contact
Kin et al 35	2003	Rat	Syngeneic pancreatic islet	Engraftment	Tissue	No	Mechanical contact
Singh et al 14	2007	Rat	Pancreas	Induced diabetes	Tissue	Yes	Mechanical contact
Singh et al 36	2008	Rat	Skin Kidney	Traumatic Ischemic	Omentum-derived stromal cells	Yes	Systemic injection
Singh et al 10	2009	Rat	Liver	Traumatic	Tissue	Yes	Mechanical contact
De Siena et al 11	2010	Pig	Myocardium	Ischemic	Omentum-derived stromal cells	No	Local injection
Zhang et al 37	2011	Rat	Scaffold for peripheral nerve	Surgical removal	Tissue	No	Mechanical contact
Mohammadi et al 38	2011	Rat	Vein graft for peripheral nerve	Surgical removal	Omentum-derived stromal cells	No	Local injection
Shah et al 15	2012	Mouse	Lung	Bleomycin	Omentum-derived stromal cells	Yes	Systemic
Bu et al 13	2014	Rat	Cornea	Alkaline	Omentum-derived stromal cells	Yes	Local injection
Garcia-Gomez et al 39	2014	Rat	Kidney	Surgical induction of chronic kidney disease	Tissue	Yes	Mechanical contact
Buyukdogan et al 40	2016	Rabbit	Cartilage	Traumatic	Tissue	No	Mechanical contact
Pinheiro et al 12	2019	Mouse	Skeletal muscle (diaphragm)	Genetic	Tissue	No	Mechanical contact
Li et al 9	2023	Mouse	Skin Peripheral nerve Peripheral vessel	Diabetic, surgical	Supernatant of cultured tissue	Yes	Local injection
Our study	2024	Mouse	Skin Soft tissue	Surgical induction of lymphedema	Supernatant of cultured tissue	Yes	Local injection

In the clinical setting, the omentum is commonly used as a pedicled flap and serves a variety of beneficial functions beyond merely filling dead spaces. Its rich vascularity and immune-modulatory properties make it an effective tool for infection control, particularly in contaminated or chronic wounds, as well as a treatment option for complex, advanced, or otherwise untreatable wounds. 19 20 The ability of the omentum to promote angiogenesis, modulate inflammation, and facilitate tissue regeneration further underscores its versatility in reconstructive and regenerative medicine. 19 21 In the context of lymphedema, the omentum is a useful therapeutic option, serving as both a lymph node donor and a source of lymphoid tissue. 22

The omentum can be activated upon exposure to foreign particles or attenuated microorganisms 6 7 and can be defined and characterized from multiple perspectives. At the gross level, the weight of the omentum substantially increases when it is activated, often reaching up to 20 times that of its native state. 6 Li et al reported that the activated omentum in an 8-week-old mouse weighs approximately 125 mg. 9 In our study, the omentum in 8-week-old mice, which received a double dose of activated omentum, weighed between 0.19 and 0.30 g. The activation process was observed at the gross level, including particle entrapment, expansion, and the formation of gross vasculature ( Fig. 1A, B ). The weight gain surpassed what might be expected from the slurry alone, suggesting that the extent of the activation can potentially be controlled by adjusting the amount of material injected.

The omental activation process has been evaluated at both the cellular and histological levels in previous studies. Electron microscopy studies revealed that activated cells predominantly comprised macrophages and mesothelial cells. 7 Litbarg et al demonstrated that the enhanced cellular components of the activated omentum are immunohistochemically positive for several stem cell markers, including *WT-1* , chemokine receptor 4, and stromal-derived factor 1 α. 6 In our study, *WT-1* -positive cells were initially widely distributed in the mesenchymal space and, due to the expansion of this mesenchymal space during the activation process, the number of *WT-1* -positive cells also increased ( Fig. 1D , not quantified). Histologically, we observed significant increases in blood vessels and their contents, further indicating the extent of omental activation. More detailed observations revealed that the primary cells involved in omental activation are characterized by distinct marker groups, namely, CD45 ^+^ /CD34 ^−^ , CD45 ^−^ /CD34 ^+^ , and CD45 ^+^ /CD34 ^−^ . These marker profiles strongly suggest that most of the activated cells are of myeloid origin. 15 A study using a parabiosis model demonstrated that most of these cells originate from circulating cells, mainly macrophages. During omental activation, these macrophages transform into fibroblast-like cells, with adipocyte progenitors playing a more limited role in this transformation. 23

The molecular characteristics of activated omentum have also been studied. The *VEGFs* are the most prominently elevated growth factor family in the activated omentum, with reported levels being approximately 3.5-fold higher than those found in native omentum across various studies. 6 Li et al, by investigating the molecular landscape of activated omentum using mass spectrometry, identified several proteins that may play a role in promoting wound healing. 9 Our immunohistochemical analyses revealed that *VEGF-C* , a well-known lymphatic-specific growth factor, was predominantly located in the interstitial spaces and subsequently released into the supernatant during the culture process ( Fig. 1C ). In our study, the *VEGF-C* level, which was first evaluated in terms of its effects on the lymphatic system, was significantly elevated in activated omentum supernatant and likely contributed to lymphatic vessel signaling pathways in vivo ( Fig. 4 ). The immunohistochemical expression of *VEGF-C* did not directly correspond to the PCR results. In its baseline or “original state,” the omentum likely maintains relatively high levels of growth factors such as *VEGF-C* , *VEGF-D* , and hepatocyte growth factor, supporting its role in immune modulation, vascularization, and tissue repair. Immunohistochemical analysis revealed that *VEGF-C* is present in the mesenchymal space, with its level increasing as the interstitial space of the omentum expands ( Fig. 1C , not quantified). 24

The genetic significance of the activation process has rarely been explored. In our study, the amount of RNA was approximately seven to eight times higher in the AO group than in the NO and PO groups, indicating a robust and ongoing activation process ( Fig. 2 ). The consistency observed between the NO and PO groups suggests that the injection of PBS did not significantly impact baseline RNA production, reaffirming the native state of the omentum. These findings indicate a substantial molecular shift upon activation that may be associated with regenerative or immune-related activity. An increase in the quantity of mRNA from whole omentum tissue indicates a temporary shift away from the maintenance of normal structure toward an active remodeling and inflammatory response ( Fig. 3A ).

The early spike in the expression of *HIF-1α* , *Ang2* , and *NRP2* by day 1 suggests an initial hypoxic and inflammatory response, which may play key roles in modulating vascular permeability and chemotaxis, particularly in glomerular-like vessels in milky spots. 25 26 27 28 Such responses recruit acute inflammatory cells in the omentum that can migrate into the peritoneal space, facilitating the eventual inoculation of foreign bodies ( Fig. 1C ). This characteristic enables the omentum to rapidly expand and provide a scaffold that supports a large accumulation of immune and reparative cells. The levels of *Foxc2* and *Prox1* increase at around days 4 and 7, promoting vascular remodeling and lymphatic adaptation. 29 30 The downregulation of *Lyve1* from day 1 suggests that the omentum is shifting from maintaining lymphatic structure to prioritizing tissue remodeling and immune cell infiltration, thereby enabling a more flexible and dynamic environment during the acute response phase. 31 32

Harvesting of the omental flap, although clinically utilized, remains an invasive procedure that is often associated with significant morbidity. In this study, we propose an alternative approach using supernatant derived from activated omental tissue. This supernatant is rich in growth factors and bioactive molecules. Our findings suggest that this material can reduce both the severity and duration of lymphedema, indicating its potential therapeutic benefit in managing conditions involving the lymphatic system ( Fig. 5 ). The supernatant may exert potent effects in the epidermis and dermis, potentially independently promoting regeneration and repair in these layers of the skin, contributing to its overall therapeutic efficacy ( Fig. 7 ). We observed less infiltration of macrophages in our AO group than in the other groups, possibly explaining the milder fibrotic changes during the lymphedema in this group; supernatant obtained from the omentum may modulate the inflammatory response, potentially limiting fibrosis during the course of lymphedema. 12

This study explores a novel approach to promoting lymphangiogenesis by investigating the role of the supernatant from the omentum in the pathogenesis of lymphedema, a condition for which effective regenerative therapies remain limited. This approach, namely, shifting from the direct application of omental tissue to a more targeted or molecular-based therapy, represents a major advance in this field.

The main limitation of this study is that our experiments were conducted in an acute phase model, which does not fully capture the chronic fibrotic changes and immune alterations observed in long-standing lymphedema. Future studies should focus on a combined graft and chronic lymphedema model to better simulate clinical conditions and provide more relevant insights into the therapeutic potential of omental tissue in long-term lymphatic regeneration.

### Conclusion

In this study, we used a mouse model to investigate the process of oocyte membrane activation and to evaluate the effects of culture supernatants derived from activated omentum on lymphangiogenesis. By examining its structural and molecular characteristics, we found that the omentum exhibits lymphoid potential. These findings highlight the capacity of activated omentum to contribute to lymphatic regeneration, providing fundamental insights into its therapeutic potential for patients with lymphedema.
